# Ultrashort Cell-Free DNA Fragments and Vimentin-Positive Circulating Tumor Cells for Predicting Early Recurrence in Patients with Biliary Tract Cancer

**DOI:** 10.3390/diagnostics14212462

**Published:** 2024-11-04

**Authors:** Sung Hee Park, Hye Ji Lee, Tae In Kim, Jonghyun Lee, Sung Yong Han, Hyung Il Seo, Dong Uk Kim

**Affiliations:** 1 Division of Gastroenterology, Biomedical Research Institute, Pusan National University Hospital, Busan 49241, Republic of Korea; scaletlee@hanmail.net (S.H.P.); lhj4151199@naver.com (H.J.L.); keiasikr@nate.com (J.L.); 2 Department of Internal Medicine, Pusan National University College of Medicine, Yangsan 44955, Republic of Korea; 3 Department of Surgery, Pusan National University College of Medicine, Yangsan 44955, Republic of Korea; seohi71@hanmail.net; 4 Department of Internal Medicine, Gumi Medical Center, CHA University, Gumi 39100, Republic of Korea; dkim74@cha.ac.kr

**Keywords:** biliary tract cancer, circulating tumor cells (CTCs), cell-free DNA (cfDNA), postoperative recurrence, recurrence-free survival (RFS)

## Abstract

**Background/Objectives:** Biliary tract cancer (BTC) is a rare but aggressive malignancy that requires surgical treatment. However, postoperative recurrence rates are high, and reliable predictors of recurrence are limited. This study aimed to investigate the effectiveness of cell-free DNA (cfDNA) and circulating tumor cells (CTCs) in predicting early recurrence after curative surgery and complete adjuvant therapy in patients with BTC. **Methods:** Twenty-four patients who underwent R0 and R1 resections and completed adjuvant therapy for BTC between September 2019 and March 2022 were followed up until March 2024. Patients were categorized into early recurrence (ER) and non-ER groups, using one year as the cutoff for recurrence. **Results:** The combination score derived from ultrashort fragments of cfDNA, vimentin-positive CTCs, and carbohydrate antigen (CA) 19-9 levels showed a statistically significant difference between the ER and non-ER groups (*p*-value < 0.001). The receiver operating characteristic curve from the combination score and CA 19-9 levels yielded areas under the curve of 0.891 and 0.750, respectively. **Conclusions:** Although further research is required, these findings suggest that cfDNA and CTCs may increase the accuracy of predicting postoperative recurrence in patients with BTC.

## 1. Introduction

Biliary tract cancer (BTC) is a rare cancer that originates from bile duct epithelial cells and has a poor prognosis. Although complete surgical resection of BTC is the only curative option, its postoperative recurrence rate remains as high as 50% [1,2]. Adjuvant treatments such as chemotherapy or radiotherapy can help prevent recurrence when pathological findings indicate a high risk of recurrence. However, many patients experience relapses despite treatment. Research to discover novel biomarkers is ongoing [3,4,5]; however, the accuracy of the prediction of postoperative recurrence remains insufficient. Postoperative carbohydrate antigen (CA) 19-9 levels have been identified as prognostic indicators in patients with BTC who have high preoperative serum CA 19-9 levels [6]. However, there are no effective circulating biomarkers for predicting early recurrence in patients with normal preoperative CA 19-9 levels.

Circulating tumor DNA (ctDNA) and circulating tumor cells (CTCs) have been actively studied as prognostic biomarkers. These circulating tumor markers have the potential to extend beyond early diagnosis to provide accurate prognoses and facilitate the discovery of novel therapeutic targets [7,8,9]. As ctDNA is derived from dying tumor cells, it is thought to be associated with a high tumor burden or rapid tumor progression. However, ctDNA is difficult to detect in patients with early-stage cancer or those with minimal residual tumors. With recent advances in ctDNA detection technology, short cell-free (cfDNA) fragments have been studied as early diagnostic markers. CTCs travel in the bloodstream through the epithelial–mesenchymal transition, which is crucial for tumor metastasis [10,11]. CTCs that exhibit mesenchymal characteristics, as indicated by vimentin positivity, are associated with cancer recurrence, progression, and metastasis. Combining ctDNA and CTC analyses may provide more accurate information regarding early tumor recurrence. However, comprehensive studies on ctDNAs and CTCs have rarely been performed.

This study aimed to investigate whether the combination of ultrashort fragments of cfDNA with vimentin-positive CTCs (vCTCs) could predict early recurrence in patients who underwent curative surgery and completed adjuvant therapy for BTC.

## 2. Materials and Methods

### 2.1. Patients

Twenty-four participants recruited from a single tertiary referral center in South Korea between September 2019 and March 2022 were followed up until June 2024. Patients with intrahepatic cholangiocarcinoma (iCCA, *n* = 2), perihilar cholangiocarcinoma (pCCA, *n* = 7), distal cholangiocarcinoma (dCCA, *n* = 10), or gallbladder cancer (GBC, *n* = 5) who underwent R0 or R1 resection were enrolled in this study. Patients with suspected BTC were evaluated preoperatively using abdominal ultrasonography, computed tomography (CT), and magnetic resonance (MR) cholangiopancreatography. This study included patients with localized tumors, who were in good general condition and who were eligible for curative resection. Patients with para-aortic lymph node metastasis, peritoneal metastasis, or liver micrometastasis discovered during abdominal examinations were excluded. Participants were divided into an early recurrence (ER) group (recurrence < 1 year postoperatively) and a non-ER group (recurrence > 1 year postoperatively or no recurrence). Blood samples were collected within 14 days after completion of curative surgery and adjuvant therapy and were preprocessed within 2 h of collection to separate plasma for cfDNA analysis and the white buffy coat for CTCs.

This study was approved by the Ethics Committee of the Pusan National University Hospital (H-H-1801-020-062) and the Clinical Research Information Service (KCT0003511).

### 2.2. cfDNA Isolation and Fragment Size Distribution Analysis

cfDNA was extracted using a QIAamp Blood Kit (Qiagen, Hilden, Germany) according to the manufacturer’s recommendations. cfDNA was extracted from 2 mL of plasma and eluted in 20 µL of elution buffer. The obtained cfDNA was subjected to a quality control test using a Nanodrop spectrophotometer (NanoDrop 2000; Thermo Fisher Scientific, Waltham, MA, USA), and samples that passed the quality control test with a cfDNA concentration of 1ng or more were used for fragment analysis. The size of cfDNA was analyzed using a high-sensitivity DNA chip and a 2100 Bioanalyzer (Agilent Technologies, Santa Clara, CA, USA) with 11 samples, 1 µL per well, on one chip. The cfDNA concentration corresponding to the base pair with the highest concentration was measured. Ultrashort and long cfDNA fragments were defined as those ranging from 90 to 150 bp and 1001 to 10,000 bp, respectively.

### 2.3. CTC Enumeration and Characterization

A commercial version of the FAST disk, the CD-PRIMETM system (Clinomics, Ulsan, Korea), was used for CTC enumeration and characterization. Intact CTCs from the white buffy coat were collected and resuspended in an equivalent volume of phosphate-buffered saline (PBS) as the original blood from each patient. Isolated cells were stained with fluorescently conjugated antibodies, including fluorescein isothiocyanate (FITC) conjugated anti-EpCAM antibody (1:417, FITC 9C4; BioLegend, San Diego, CA, USA), Alexa 488 conjugated anti-pan-cytokeratin antibody (1:100, AE1/AE3; Invitrogen, Carlsbad, CA, USA), Alexa 555-conjugated anti-vimentin antibody (1:125, D21H3; Cell signaling, Danvers, MA, USA), and Alexa 647-conjugated anti-CD45 antibody (1:50, H130; BioLegend, San Diego, CA, USA). Total CTC and vimentin-positive CTC (vCTC) counts were determined according to methods described in a previous paper [10].

### 2.4. Outcome Assessment

The primary endpoint was to determine whether the combination of ultrashort cfDNA fragments and vCTCs correlated with early postoperative recurrence (<1 year after curative surgery). The secondary endpoint was the correlation between the total CTC count and cfDNA fragment concentration. Patients were followed up for disease progression using imaging and laboratory tests. Recurrence-free survival (RFS) was defined as the duration from surgery to relapse and assessed using peripheral blood sample collection, CT and MRI scans, and carbohydrate antigen 19-9 (CA19-9) levels. Overall survival (OS) was defined as the time from pathological diagnosis to death.

### 2.5. Statistical Analysis

Statistical analyses were performed using the IBM SPSS statistical software (version 21.0; IBM Corp., Armonk, NY, USA). The descriptive statistics were expressed as frequencies and percentages for categorical variables and as means ± standard deviations for continuous variables. For normally distributed variables, Fisher’s exact test was used to compare two samples. For nonparametric comparisons, the Wilcoxon rank-sum and Kruskal–Wallis tests were used. In all analyses, a two-sided *p*-value <0.05 was used to indicate statistical significance. The optimal cut-off values for the cfDNA and CTC variables were determined using receiver operating characteristic (ROC) curves, and the area under the curve (AUC) was calculated. Differences in OS were displayed using Kaplan–Meier survival plots and tested using the log-rank test. To evaluate factors affecting prognosis, COX regression analysis was performed using cfDNA and CTC as known prognostic markers.

## 3. Results

### 3.1. Patient Characteristics

The mean age of all patients with BTC (62.5% male) was 67.4 years. The incidence of nodal metastasis differed significantly between the ER and non-ER groups (100% vs. 37.5 %, *p* = 0.004). Neither R0 resection nor adjuvant therapy was associated with early recurrence. The Carcino-Embryonic Antigen (CEA) (3.33 vs. 2.41 ng/mL, *p* = 0.221) and CA19-9 (90.8 vs. 70.1 U/mL, *p* = 0.209) levels were not significantly different between the ER and non-ER groups. All epidemiological and laboratory factors were similar in both groups (Table 1).

### 3.2. cfDNA Fragments and CTC Counts Between ER and Non-ER Groups

The concentration (858.2 vs. 173.7 pg/µL, *p* < 0.001) and proportion (31.0 vs. 15.7%, *p* < 0.001) of ultrashort cfDNA fragments were significantly higher in patients who experienced early recurrence than in patients without early recurrence. Furthermore, although the proportion of vCTCs was higher in the ER group than in the non-ER group, the difference was not significant. In contrast, the vCTC counts were significantly different between the groups (48.3 vs. 25.8, *p* = 0.010) (Table 1).

### 3.3. Combination Score for Predicting Early Recurrence

**Figure 1:**
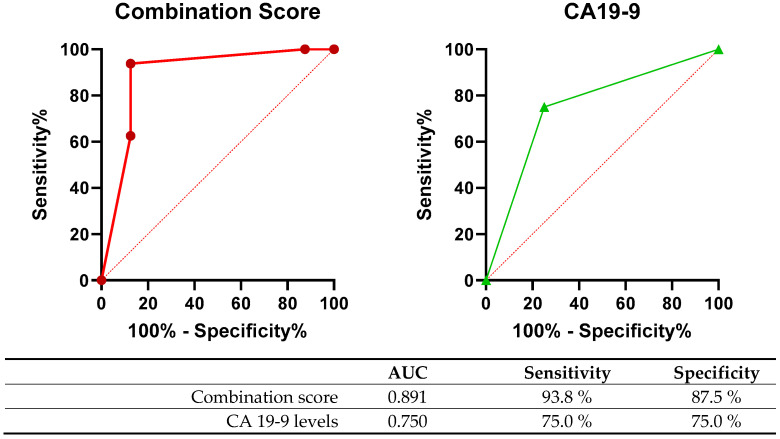
ROC curve with combination score, CA 19-9 levels, and ultrashort cfDNA fragments for early recurrence (AUC: area under curve).

The cut-off values for blood biomarkers that differed significantly between the two groups were established using ROC curve analysis. The key factors identified in the univariate analysis included ultrashort cfDNA fragment concentrations > 169.8 pg/µL along with a proportion of ultrashort cfDNA fragments > 15.1%, vCTC counts > 15/mL with a vCTC proportion > 40%, and CA 19-9 levels > 39 U/mL. To prepare a clinically applicable combination score, each significant factor in the univariate analysis was assigned one point (Table 2). The combined scoring system demonstrated sensitivity of 93.8% and specificity of 87.5% for evaluating patients. As observed from the wider AUC in the ROC curve analysis, the combination score predicted the possibility of recurrence more accurately than the CA 19-9 levels (Figure 1).

### 3.4. Association of Combination Score with Prognosis

**Figure 2:**
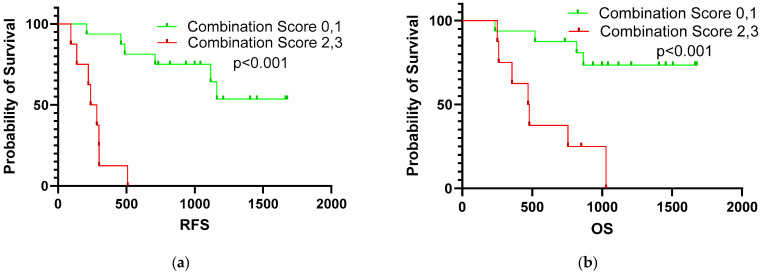
Survival curve of combination score for RFS (**a**) and OS (**b**).

The combination score showed the most significant prognostic value (Figure 2). Among patients with BTC, those with a combination score of 2–3 had a worse prognosis than those with other scores (median survival: undefined (209–1677) vs. 261 (94–509) days, respectively; *p* < 0.001). Patients with BTC exhibited comparable median OS rates between the 0 and 1 and 2–3 combination score groups (OS, undefined (235–1677) vs. 475 (250–1028) days; *p* < 0.001).

### 3.5. Correlation Between CTC Counts and Fragment Length of cfDNA

**Figure 3:**
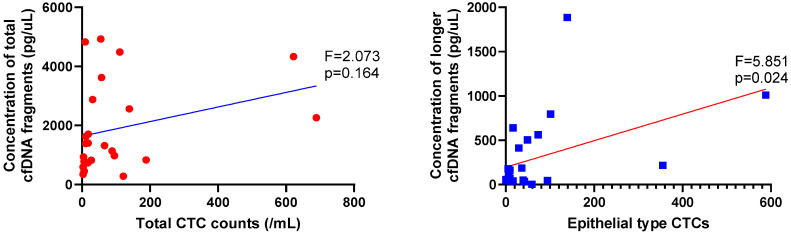
Epithelial-type CTCs correlated with the concentration of longer cfDNA fragments.

The total CTC counts did not show a linear correlation with the total cfDNA concentration (Figure 3). There was a negative correlation between vCTC count and the concentration of ultrashort cfDNA fragments; however, this was not statistically significant. Epithelial-type CTCs were significantly correlated with the concentration of longer cfDNA fragments (F = 5.851, *p* = 0.024).

## 4. Discussion

The combined score of ultrashort cfDNA fragments, vCTCs, and CA 19-9 levels was more accurate in predicting early postoperative recurrence than CA 19-9 levels alone in patients with BTC following complete adjuvant therapy.

In previous studies, the most powerful factors predicting postoperative recurrence were largely based on surgical pathological findings. Adjuvant chemotherapy was administered based on predictive pathological factors, such as cancer invasion, lymph node metastasis, lymphovascular invasion, perineural invasion, and R1 resection [12,13]. However, these factors require the acquisition of surgical specimens, and it is uncertain whether these postoperative pathological findings are strong predictors after adjuvant chemotherapy or radiotherapy. In many cases, the risk of recurrence remains high despite adjuvant treatment. In such cases, additional adjuvant treatment can be administered for extended periods (≥1 year). However, this strategy cannot be applied to all patients because of the complications associated with chemotherapy and the potential decline in quality of life. Therefore, more refined biomarkers are required for personalized adjuvant therapy.

Taiichi et al. studied the utility of an artificial intelligence deep learning model to predict early postoperative recurrence in patients with iCCA using CT images [14]. Although the model exhibited a high accuracy of 96.5% on the validation dataset, it was limited to the iCCA. Furthermore, CA 19-9 was identified as a strong predictor of recurrence if its concentration was increased in patients with high preoperative CA 19-9 levels [6]. However, this finding was not statistically significant in patients with normal CA 19-9 levels. Therefore, identifying a blood biomarker to predict postoperative recurrence is crucial because it is less invasive and allows for repeated acquisition.

Recently, with the development of analytical techniques, many studies have used cfDNA and CTCs in the blood to predict postoperative recurrence. The presence of ctDNA and mesenchymal CTCs after the completion of curative surgery and adjuvant therapy is associated with the early recurrence of various gastrointestinal malignancies [15,16,17,18]. Yu et al. reported an increased ctDNA titer in an 81-year-old male with stage III iCCA who received adjuvant chemotherapy after curative surgery. Additionally, his CA19-9 levels increased, but there was no evidence of recurrence on the CT scan. Therefore, considering the MSI-H status of the tumor, the adjuvant chemotherapy was switched from capecitabine to pembrolizumab. Subsequently, ctDNA became undetectable, and the CA19-9 levels returned to the reference range following pembrolizumab treatment. This case demonstrates the potential utility of tumor-informative ctDNA in CCA [19]. Wang et al. reported that plasma cfDNA copy number variation (CNV) signals are strong in BTC, and their sensitivity and specificity are higher than those of CA19-9, suggesting its usefulness for clinical diagnosis [20]. Recently, ctDNA in the peripheral blood of patients with biliary tract cancer has shown great potential for application at every stage of cancer management, including early cancer diagnosis, the identification of driver mutations, the monitoring of treatment response, and the detection of resistance mechanisms, suggesting its great clinical utility [21]. However, because ctDNA detection requires considerable effort and cost, a simpler and cheaper technique is required to detect changes in ctDNA titers.

The characterization of cfDNA fragments mainly focuses on fragment length distribution, which is easier and cheaper than next-generation sequencing analysis [22,23,24]. A study on breast cancer reported that a higher ratio of longer fragments to total DNA may be clinically useful for detecting breast cancer progression [24]. The higher the ratio of longer fragments, the more advanced the tumor stage and LN metastasis. This finding suggests that larger tumors lead to increased cfDNA secretion due to necrosis, resulting in longer cfDNA fragments. However, ctDNA is believed to contain shorter fragments because histone binding is looser in ctDNA than in normal cfDNA. This preliminary study on breast cancer did not include ctDNAs smaller than 115 bp; therefore, it is difficult to view it as a study reflecting ctDNAs smaller than 150 bp. In contrast, some studies have noted that shorter amplicons of 60–100 bp are optimal for quantifying ctDNA because longer amplicons significantly estimate ctDNA levels [25,26]. When the fragment size of the plasma ctDNA was between 90 and 150 bp, it was possible to classify high-ctDNA cancers, such as BTC, in more than 95% of cases. ctDNA is best identified through enrichment with ultrashort cfDNA fragments ranging from 90 to 150 bp [27,28,29,30]. Therefore, identifying ultrashort cfDNA fragments may facilitate noninvasive cancer detection and monitoring.

In this study, both the absolute concentration of ultrashort cfDNA fragments and the proportion of ultrashort cfDNA fragments were significantly associated with early recurrence. This is consistent with the results of a previous study that showed that ultrashort cfDNA fragments contain more ctDNA [28,31,32]. In one study, a CLAmp-seq analysis method was developed to analyze single-stranded ultrashort ctDNA of ˂100 bp, which was able to preserve and analyze single-stranded ultrashort ctDNA carrying unique cancer signals that may be missed by double-stranded DNA. Ultrashort ctDNA fragments show a high sensitivity of 60% and a specificity of 98% in various patients with cancer, suggesting that they can detect cancer mutations and enable early diagnosis [33]. Mouliere et al. reported that the concentration of tumor-derived ctDNA fragments was positively correlated with tumor burden. The quantification of ctDNA by qPCR was optimal for ultrashort fragments of 60–100 bp. Patients with metastatic colorectal cancer had nearly 5 times higher mean ctDNA fragmentation than healthy individuals. The fragmentation of cfDNA is higher after apoptosis than after necrosis or phagocytosis [23]. cfDNA fragments shorter than 1000 bp, particularly of 180 bp, are reminiscent of the oligonucleosomal DNA ladder observed in apoptotic cells [24]. By distinguishing the fragment size of cfDNA, it is possible to determine whether cfDNA originates mainly from apoptotic cancer cells and whether residual cancer cells are present.

In this study, the number of vCTCs and the proportion of vCTCs relative to the total number of CTCs were higher in the ER group. vCTCs are suitable candidates as diagnostic and prognostic biomarkers; however, there are limited studies on this topic. Our research team conducted a preliminary study to explore the utility of vCTCs in distinguishing patients with BTC from those with benign biliary tract disease (BBD) [10]. A vCTC count > 15/mL of blood (57.7% vs. 10%, *p* = 0.005) and a vCTC/CTC ratio > 40% (48.1% vs. 10%, *p* = 0.025) demonstrated significant differences between BTC and BBD, suggesting that vCTCs may serve as potential biomarkers for early diagnosis and prognosis prediction in patients with BTC.

CTCs circulate in the bloodstream through epithelial–mesenchymal processes, which play an important role in tumor metastasis. Although tumor cells can be detected in healthy individuals, their diagnostic value remains unclear. However, metastasis and tumor progression play important roles in cases involving tumor cells with mesenchymal chromatin, suggesting that they may be useful for identifying minimal residual disease. In this study, there was no difference in the total CTC count between the early relapse and non-relapse groups; however, there was a difference in vCTC counts [34,35].

In malignant diseases without representative prognostic biomarkers, scoring systems are often used to predict tumor progression. Intraductal papillary mucinous neoplasia (IPMN) of the pancreas is a representative disease that uses a scoring system to improve malignancy prediction accuracy [36]. In this study, a combination score was developed by integrating the main predictive factors, cfDNA and CTCs, which reflect various cancer biologies. Although ctDNAs and CTCs were not present in any of the patient samples, they were occasionally detected in benign patients without cancer. Therefore, testing both ctDNAs and CTCs in tumor-derived blood samples may lead to the identification of additional tumor-specific biomarkers. Furthermore, the simultaneous detection of ctDNA and CTCs improves the accuracy of tumor diagnosis and progression assessment. A cut-off value was established using the ROC curve analysis for short cfDNA fragments, vCTCs, and CA 19-9 levels. Each value exceeding the cutoff was assigned 1 point, resulting in a score ranging from 0 to 3 points. In cases with a combination score of two or three points, the AUC value for predicting recurrence was high, whereas the PFS and OS were poor. Ningyuan et al. evaluated scoring systems using triple serum exosomal circular RNA from patients with CCA and bile duct obstruction and confirmed its association with early postoperative recurrence [37]. The diagnostic ability of the serum exosomal circRNA signature (serum DS, AUROC = 0.861, RR = 4.04) was superior to that of the conventional CA19-9 signature (AUROC = 0.759, RR = 2.08). Researchers calculated the early recurrence score to establish a prognostic model for patients with CCA undergoing curative surgery and combined this score with other prognostic factors to establish a nomogram for monitoring CCA recurrence.

CTCs and cfDNA are known to have different or similar characteristics, depending on the biology of the tumor. In this study, the absolute concentration of longer cfDNA fragments positively correlated with the number of epithelial-type CTCs. DNA longer than 1000 bp is thought to result from cell necrosis. As tumor volume increases, more DNA is produced through necrosis, leading to the presence of larger cfDNA fragments [20]. Similarly, larger tumors with greater vascular invasion are likely to release more epithelial CTCs into the blood [38,39]. Therefore, longer cfDNA fragments are believed to positively correlate with epithelial CTCs.

Although ctDNAs and CTCs are thought to help diagnose tumors and predict their prognosis, there is no established strategy for interpreting tumor characteristics. This study focused on patients diagnosed with biliary tract cancer who underwent surgery at our center over approximately 2 years and 6 months, which inevitably resulted in a small sample size. This is one of the limitations of our study and a key reason for planning further validation studies. Because the number of patients in this study was small, each factor may have been interpreted arbitrarily, which may have led to selection bias. However, if the correlation with clinical course data is properly interpreted, it is expected that it will be able to reflect both the characteristics of primary tumors and metastatic lesions, including the biological factors and tumor heterogeneity. This study analyzed all heterogeneous BTCs, including iCCAs, GBCs, and eCCAs, which have different prognoses and recurrence patterns. Although cfDNA and CTCs may show different cut-off values depending on the type of BTC, they are expected to show a consistent cut-off value regardless of the recurrence pattern according to the cancer type after complete surgery and adjuvant therapy. Therefore, ctDNAs and CTCs are considered useful biomarkers for predicting recurrence. Furthermore, research analyzing these genes or tumor cells is expected to increase in the future.

## 5. Conclusions

The combined score of 3, calculated by combining short fragments of cfDNA, vCTCs, and CA 19-9 levels, exhibited a very high predictive value (AUC = 0.891) for recurrence within 1 year. Although further studies are required, this study suggests that cfDNA and CTCs may increase the accuracy of predicting recurrence after the completion of adjuvant chemotherapy following curative surgery in patients with BTC.

## Figures and Tables

**Table 1:** Patient characteristics in ER and non-ER groups.

	BTC (*n* = 24)		*p*-Value
	ER (*n* = 8)	Non-ER (*n* = 16)	
Sex male	7 (87.5)	8 (50.0)	0.087
Age, mean ± SD	68.6 ± 7.2	66.8 ± 10.8	0.673
Diagnosis (dCCA/pCCA/iCCA/GBC)	3 (37.5)/3 (37.5)/0 (0)/2 (25.0)	7 (43.75)/4 (25.0)/2 (12.5)/3 (18.75)	0.697
Viral hepatitis	0 (0)	0 (0)	-
Clonorchiasis Hx	1 (12.5)	3 (18.75)	0.593
Hypertension	5 (62.5)	8 (50.0)	0.444
Diabetes	3 (37.5)	3 (18.75)	0.302
Smoker	2 (25.0)	3 (18.75)	0.555
Alcohol consumption	3 (37.5)	2 (12.5)	0.186
BMI, mean ± SD	25.3 ± 2.3	23.4 ± 3.3	0.158
**Surgical pathologic findings**			
R0/R1 resection	4 (50.0)/4 (50.0)	14 (87.5)/2 (12.5)	0.069
Node metastasis	8 (100)	6 (37.5)	0.004
Adjuvant chemotherapy Capecitabine/FLv/Gemcitabine/CCRTx	1 (12.5)/3 (37.5)/1 (12.5)/3 (37.5)	4 (25.0)/5 (31.3)/1 (6.2)/6 (37.5)	0.445
**Laboratory findings**			
WBC (/µL)	5221.7 ± 1985.9	5385.0 ± 1904.3	0.885
NLR	78.7 ± 140.2	68.4 ± 131.1	0.861
Hb (g/dL)	12.3 ± 1.6	11.9 ± 1.3	0.436
PLT (k/µL))	199.3 ± 101.4	203.6 ± 84.4	0.913
ALT (U/L)	17.0 ± 6.4	22.6 ± 17.6	0.396
ALP (U/L)	90.9 ± 38.6	102.4 ± 30.5	0.431
Total Bilirubin (g/dL)	0.59 ± 0.42	0.52 ± 0.30	0.637
Albumin (g/dL)	4.00 ± 0.62	4.22 ± 0.46	0.333
CEA (ng/mL)	3.33 ± 1.71	2.41 ± 1.49	0.221
CA19-9 (U/mL)	90.8 ± 106.2	70.1 ± 170.7	0.209
**Liquid biopsy findings**			
Total cfDNA (pg/µL)	2530.1 ± 1839.2	1562.5 ± 1244.8	0.140
UF of cfDNA (pg/µL)	858.2 ± 1380.6	173.7 ± 66.7	<0.001
UF proportion of cfDNA (%)	31.0 ± 30.4	15.7 ± 8.0	<0.001
LF of cfDNA (pg/µL)	271.3 ± 238.1	266.1 ± 369.2	0.244
LF proportion of cfDNA (%)	16.0 ± 25.5	15.4 ± 14.4	0.060
Total CTC count (/mL)	128.4 ± 204.6	85.5 ± 169.7	0.513
vCTC count (/mL)	48.3 ± 91.5	25.8 ± 41.6	0.010
vCTC proportion (%)	27.0 ± 18.1	39.6 ± 24.7	0.413

BTC (biliary tract cancer), dCCA (distal cholangiocarcinoma), pCCA (perihilar cholangiocarcinoma), iCCA (intrahepatic cholangiocarcinoma), GBC (gallbladder cancer), NLR (neutrophil-to-lymphocyte ratio), vCTC (vimentin-positive CTC), vCTC proportion of (vCTC/total CTC ratio), UF (ultrashort fragments; 90–150 bp length of cfDNA), LF (longer fragments; 1001–10,000 bp length of cfDNA).

**Table 2:** CTC count, length of cfDNA fragment, and combination score between the ER and non-ER groups.

	BTC (*n* = 24)		*p*-Value	Acquired Point
	ER (*n* = 8)	Non-ER (*n* = 16)		
Total cfDNA > 1515 pg/µL	5	5	0.153	
UF of cfDNA > 169.8 pg/µL and UF proportion of cfDNA > 15.1%	4	1	0.028	YES, 1; No, 0
LF of cfDNA < 101.2 pg/µL and LF proportion of cfDNA < 12.6%	2	9	0.156	
Total CTC count > 40/mL	4	7	0.556	
vCTC count > 15/mL and vCTC proportion > 40%	5	2	0.021	YES, 1; No, 0
CA 19-9 levels > 39 U/mL	6	4	0.028	YES, 1; No, 0
**Combination score**	**1.9 ± 0.8**	**0.4 ± 0.6**	**<0.001**	
**0.1 vs. 2.3**	**1/7**	**15/1**	**<0.001**	

BTC (biliary tract cancer), vCTC (vimentin-positive circulating tumor cells), vCTC proportion (vCTC/total CTC ratio), UF (ultrashort fragments; 90–150 bp length of cfDNA), and LF (longer fragments; 1001–10,000 bp length of cfDNA).

## Data Availability

Data pertaining to this article will be shared upon reasonable request with the corresponding author.

## References

[1] Razumilava N., Gores G.J. (2014). Cholangiocarcinoma. Lancet.

[2] Sakata J., Nomura T., Aono T., Kitami C., Yokoyama N., Minagawa M., Takizawa K., Miura K., Hirose Y., Ichikawa H. (2021). Oncological outcomes of surgery for recurrent biliary tract cancer: Who are the best candidates?. HPB.

[3] Chan-On W., Nairismägi M.L., Ong C.K., Lim W.K., Dima S., Pairojkul C., Lim K.H., McPherson J.R., Cutcutache I., Heng H.L. (2013). Exome sequencing identifies distinct mutational patterns in liver fluke-related and non-infection-related bile duct cancers. Nat. Genet..

[4] Jiao Y., Pawlik T.M., Anders R.A., Selaru F.M., Streppel M.M., Lucas D.J., Niknafs N., Guthrie V.B., Maitra A., Argani P. (2013). Exome sequencing identifies frequent inactivating mutations in BAP1, ARID1A and PBRM1 in intrahepatic cholangiocarcinomas. Nat. Genet..

[5] Ong C.K., Subimerb C., Pairojkul C., Wongkham S., Cutcutache I., Yu W., McPherson J.R., Allen G.E., Ng C.C., Wong B.H. (2012). Exome sequencing of liver fluke-associated cholangiocarcinoma. Nat. Genet..

[6] Kato Y., Takahashi S., Gotohda N., Konishi M. (2016). Prognostic Impact of the Initial Postoperative CA19-9 Level in Patients with Extrahepatic Bile Duct Cancer. J. Gastrointest. Surg..

[7] Bernard V., Kim D.U., San Lucas F.A., Castillo J., Allenson K., Mulu F.C., Stephens B.M., Huang J., Semaan A., Guerrero P.A. (2019). Circulating Nucleic Acids Are Associated With Outcomes of Patients With Pancreatic Cancer. Gastroenterology.

[8] Cayrefourcq L., Mazard T., Joosse S., Solassol J., Ramos J., Assenat E., Schumacher U., Costes V., Maudelonde T., Pantel K. (2015). Establishment and characterization of a cell line from human circulating colon cancer cells. Cancer Res..

[9] Maheswaran S., Haber D.A. (2015). Ex Vivo Culture of CTCs: An Emerging Resource to Guide Cancer Therapy. Cancer Res..

[10] Han S.Y., Park S.H., Ko H.S., Jang A., Seo H.I., Lee S.J., Kim G.H., Kim D.U. (2021). Vimentin-Positive Circulating Tumor Cells as Diagnostic and Prognostic Biomarkers in Patients with Biliary Tract Cancer. J. Clin. Med..

[11] Semaan A., Bernard V., Kim D.U., Lee J.J., Huang J., Kamyabi N., Stephens B.M., Qiao W., Varadhachary G.R., Katz M.H. (2021). Characterisation of circulating tumour cell phenotypes identifies a partial-EMT sub-population for clinical stratification of pancreatic cancer. Br. J. Cancer.

[12] Akita M., Ajiki T., Ueno K., Tsugawa D., Hashimoto Y., Tanaka M., Kido M., Toyama H., Fukumoto T. (2020). Predictors of postoperative early recurrence of extrahepatic bile duct cancer. Surg. Today.

[13] Ito Y., Abe Y., Egawa T., Kitago M., Itano O., Kitagawa Y. (2018). Predictive Factors of Early Recurrence in Patients with Distal Cholangiocarcinoma after Pancreaticoduodenectomy. Gastroenterol. Res. Pract..

[14] Wakiya T., Ishido K., Kimura N., Nagase H., Kanda T., Ichiyama S., Soma K., Matsuzaka M., Sasaki Y., Kubota S. (2022). CT-based deep learning enables early postoperative recurrence prediction for intrahepatic cholangiocarcinoma. Sci. Rep..

[15] Azad T.D., Chaudhuri A.A., Fang P., Qiao Y., Esfahani M.S., Chabon J.J., Hamilton E.G., Yang Y.D., Lovejoy A., Newman A.M. (2020). Circulating Tumor DNA Analysis for Detection of Minimal Residual Disease After Chemoradiotherapy for Localized Esophageal Cancer. Gastroenterology.

[16] Lin D., Shen L., Luo M., Zhang K., Li J., Yang Q., Zhu F., Zhou D., Zheng S., Chen Y. (2021). Circulating tumor cells: Biology and clinical significance. Signal Transduct. Target. Ther..

[17] Reinert T., Henriksen T.V., Christensen E., Sharma S., Salari R., Sethi H., Knudsen M., Nordentoft I., Wu H.T., Tin A.S. (2019). Analysis of Plasma Cell-Free DNA by Ultradeep Sequencing in Patients With Stages I to III Colorectal Cancer. JAMA Oncol..

[18] Yang J., Gong Y., Lam V.K., Shi Y., Guan Y., Zhang Y., Ji L., Chen Y., Zhao Y., Qian F. (2020). Deep sequencing of circulating tumor DNA detects molecular residual disease and predicts recurrence in gastric cancer. Cell Death Dis..

[19] Yu J., Avriett T.A., Ray C.M., Kim R.D. (2024). Circulating tumor DNA analysis guiding adjuvant treatment in resected stage III cholangiocarcinoma: A case report. J. Gastrointest. Oncol..

[20] Wang X., Fu X.H., Qian Z.L., Zhao T., Duan A.Q., Ruan X., Zhu B., Yin L., Zhang Y.J., Yu W.L. (2021). Non-invasive detection of biliary tract cancer by low-coverage whole genome sequencing from plasma cell-free DNA: A prospective cohort study. Transl. Oncol..

[21] Rizzo A., Ricci A.D., Tavolari S., Brandi G. (2020). Circulating Tumor DNA in Biliary Tract Cancer: Current Evidence and Future Perspectives. Cancer Genom. Proteom..

[22] Chan K.C., Zhang J., Hui A.B., Wong N., Lau T.K., Leung T.N., Lo K.W., Huang D.W., Lo Y.M. (2004). Size distributions of maternal and fetal DNA in maternal plasma. Clin. Chem..

[23] Jahr S., Hentze H., Englisch S., Hardt D., Fackelmayer F.O., Hesch R.D., Knippers R. (2001). DNA fragments in the blood plasma of cancer patients: Quantitations and evidence for their origin from apoptotic and necrotic cells. Cancer Res..

[24] Umetani N., Giuliano A.E., Hiramatsu S.H., Amersi F., Nakagawa T., Martino S., Hoon D.S. (2006). Prediction of breast tumor progression by integrity of free circulating DNA in serum. J. Clin. Oncol..

[25] Bhambhani C., Kang Q., Hovelson D.H., Sandford E., Olesnavich M., Dermody S.M., Wolfgang J., Tuck K.L., Brummel C., Bhangale A.D. (2024). ctDNA transiting into urine is ultrashort and facilitates noninvasive liquid biopsy of HPV+ oropharyngeal cancer. JCI Insight.

[26] Li F., Wei F., Huang W.L., Lin C.C., Li L., Shen M.M., Yan Q., Liao W., Chia D., Tu M. (2020). Ultra-Short Circulating Tumor DNA (usctDNA) in Plasma and Saliva of Non-Small Cell Lung Cancer (NSCLC) Patients. Cancers.

[27] Mouliere F., Chandrananda D., Piskorz A.M., Moore E.K., Morris J., Ahlborn L.B., Mair R., Goranova T., Marass F., Heider K. (2018). Enhanced detection of circulating tumor DNA by fragment size analysis. Sci. Transl. Med..

[28] Phallen J., Sausen M., Adleff V., Leal A., Hruban C., White J., Anagnostou V., Fiksel J., Cristiano S., Papp E. (2017). Direct detection of early-stage cancers using circulating tumor DNA. Sci. Transl. Med..

[29] Chen E., Cario C.L., Leong L., Lopez K., Márquez C.P., Chu C., Li P.S., Oropeza E., Tenggara I., Cowan J. (2021). Cell-free DNA concentration and fragment size as a biomarker for prostate cancer. Sci. Rep..

[30] Underhill H.R., Kitzman J.O., Hellwig S., Welker N.C., Daza R., Baker D.N., Gligorich K.M., Rostomily R.C., Bronner M.P., Shendure J. (2016). Fragment Length of Circulating Tumor DNA. PLoS Genet..

[31] Mouliere F., Robert B., Arnau Peyrotte E., Del Rio M., Ychou M., Molina F., Gongora C., Thierry A.R. (2011). High fragmentation characterizes tumour-derived circulating DNA. PLoS ONE.

[32] Strickler J.H., Loree J.M., Ahronian L.G., Parikh A.R., Niedzwiecki D., Pereira A.A.L., McKinney M., Korn W.M., Atreya C.E., Banks K.C. (2018). Genomic Landscape of Cell-Free DNA in Patients with Colorectal Cancer. Cancer Discov..

[33] Wang F., Li X., Li M., Liu W., Lu L., Li Y., Chen X., Yang S., Liu T., Cheng W. (2024). Ultra-short cell-free DNA fragments enhance cancer early detection in a multi-analyte blood test combining mutation, protein and fragmentomics. Clin. Chem. Lab. Med..

[34] Jie X.X., Zhang X.Y., Xu C.J. (2017). Epithelial-to-mesenchymal transition, circulating tumor cells and cancer metastasis: Mechanisms and clinical applications. Oncotarget.

[35] Yang J., Weinberg R.A. (2008). Epithelial-mesenchymal transition: At the crossroads of development and tumor metastasis. Dev. Cell.

[36] Shin S.H., Han D.J., Park K.T., Kim Y.H., Park J.B., Kim S.C. (2010). Validating a simple scoring system to predict malignancy and invasiveness of intraductal papillary mucinous neoplasms of the pancreas. World J. Surg..

[37] Wen N., Peng D., Xiong X., Liu G., Nie G., Wang Y., Xu J., Wang S., Yang S., Tian Y. (2024). Cholangiocarcinoma combined with biliary obstruction: An exosomal circRNA signature for diagnosis and early recurrence monitoring. Signal Transduct. Target. Ther..

[38] Anker P., Mulcahy H., Chen X.Q., Stroun M. (1999). Detection of circulating tumour DNA in the blood (plasma/serum) of cancer patients. Cancer Metastasis Rev..

[39] Sorenson G.D., Pribish D.M., Valone F.H., Memoli V.A., Bzik D.J., Yao S.L. (1994). Soluble normal and mutated DNA sequences from single-copy genes in human blood. Cancer Epidemiol. Biomark. Prev..

